# Acute Anxiety Predicts Components of the Cold Shock Response on Cold Water Immersion: Toward an Integrated Psychophysiological Model of Acute Cold Water Survival

**DOI:** 10.3389/fpsyg.2018.00510

**Published:** 2018-04-11

**Authors:** Martin J. Barwood, Jo Corbett, Heather Massey, Terry McMorris, Mike Tipton, Christopher R. D. Wagstaff

**Affiliations:** ^1^Department of Sport, Health and Nutrition, Leeds Trinity University, Leeds, United Kingdom; ^2^Department of Sport and Exercise Science, University of Portsmouth, Portsmouth, United Kingdom; ^3^Department of Psychology, Faculty of Health and Life Sciences, Northumbria University, Newcastle upon Tyne, United Kingdom

**Keywords:** drowning prevention, cold water, “float first”, cold-water survival, open water safety

## Abstract

**Introduction:** Drowning is a leading cause of accidental death. In cold-water, sudden skin cooling triggers the life-threatening cold shock response (CSR). The CSR comprises tachycardia, peripheral vasoconstriction, hypertension, inspiratory gasp, and hyperventilation with the hyperventilatory component inducing hypocapnia and increasing risk of aspirating water to the lungs. Some CSR components can be reduced by habituation (i.e., reduced response to stimulus of same magnitude) induced by 3–5 short cold-water immersions (CWI). However, high levels of acute anxiety, a plausible emotion on CWI: magnifies the CSR in unhabituated participants, reverses habituated components of the CSR and prevents/delays habituation when high levels of anxiety are experienced concurrent to immersions suggesting anxiety is integral to the CSR.

**Purpose:** To examine the predictive relationship that *prior* ratings of acute anxiety have with the CSR. Secondly, to examine whether anxiety ratings correlated with components of the CSR *during* immersion before and after induction of habituation.

**Methods:** Forty-eight unhabituated participants completed one (CON1) 7-min immersion in to cold water (15°C). Of that cohort, twenty-five completed four further CWIs that would ordinarily induce CSR habituation. They then completed two counter-balanced immersions where anxiety levels were increased (CWI-ANX) or were not manipulated (CON2). Acute anxiety and the cardiorespiratory responses (cardiac frequency [*f*_c_], respiratory frequency [*f*_R_], tidal volume [*V_T_*], minute ventilation [*
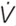
*_E_]) were measured. Multiple regression was used to identify components of the CSR from the most life-threatening period of immersion (1^st^ minute) predicted by the anxiety rating *prior to* immersion. Relationships between anxiety rating and CSR components *during* immersion were assessed by correlation.

**Results:** Anxiety rating predicted the *f*_c_ component of the CSR in unhabituated participants (CON1; *p* < 0.05, *r* = 0.536, *r*^2^= 0.190). After habituation immersions (i.e., cohort 2), anxiety rating predicted the *f*_R_ component of the CSR when anxiety levels were lowered (CON2; *p* < 0.05, *r* = 0.566, *r*^2^= 0.320) but predicted the *f*_c_ component of the CSR (*p* < 0.05, *r* = 0.518, *r*^2^= 0.197) when anxiety was increased suggesting different drivers of the CSR when anxiety levels were manipulated; correlation data supported these relationships.

**Discussion:** Acute anxiety is integral to the CSR before and after habituation. We offer a new integrated model including neuroanatomical, perceptual and attentional components of the CSR to explain these data.

## Introduction

A conservative estimate suggests that approximately 375,000 people unintentionally enter in to water and drown each year (World Health Organization, 2014) although the true figure may be four or five times higher (Bierens et al., 2016). Consequently, death by drowning is the second most common cause of accidental death in adults and the third most common cause in children in most countries (Bierens et al., 2002). If the water is cold, the physiological responses evoked during the first few minutes of whole body cold water immersion (CWI) are life threatening (Tipton, 2003) and are strongly implicated in this drowning statistic (Tipton, 1989) even in strong swimmers or those with basic survival skills (Golden et al., 1986; Bowes et al., 2016). The responses evoked by CWI, known collectively as the cold shock response (CSR Tipton, 1989), include an “inspiratory gasp,” (Burke and Mekjavic, 1991) hyperventilation, a resultant hypocapnia, tachycardia, peripheral vasoconstriction and hypertension (Keatinge and Nadel, 1965; Cooper et al., 1976). The hyperventilatory component of the CSR significantly decreases maximum breath hold time in the majority of participants, thus increasing the chances of involuntarily aspirating water and drowning (Hayward and French, 1989; Tipton and Vincent, 1989); this represents a further hazard to that posed by the high cardiovascular strain (Tipton et al., 1994). The current behavioral recommendation to survive acute accidental CWI is to “float first” (Barwood et al., 2011) and for those who are unable to float without aid to “float first and kick for your life” thereby providing some further buoyancy (Barwood et al., 2016). The CSR subsides after the initial peak at 60–90 s and swimming to safe refuge or executing a survival strategy may become possible (Golden et al., 1986; Bowes et al., 2016).

For those at risk individuals (e.g., pilots, naval personnel, persons recreating on/near water), protective steps should be taken to reduce the magnitude of CSR on water entry. For example protective clothing may mitigate the rapid skin cooling that evokes the CSR (Tipton, 1991; Power et al., 2016) but this is not always feasible, especially if immersion is unexpected. An alternative may be to induce an habituation of the CSR which can be achieved by undergoing a minimum of four short CWIs following which the CSR is significantly blunted (Tipton et al., 1998; Barwood et al., 2007); habituation is defined as reduced response to a stimulus of the same magnitude (Zald, 2003). The consequence of habituation is a significantly reduced cardiorespiratory response to CWI which may be retained for 7 months and partially present up to 14 months later (Tipton et al., 2000). Reducing the CSR may confer some benefit to defending the airway in the emergency scenario as the hyperventilatory drive seen in unhabituated participants is significantly reduced (Tipton et al., 1998), although large variability in the habituation of the response is often seen (Barwood et al., 2007).

Part of the variability between individuals in the CSR could be accounted for by differences in psychological state both prior to, and during a CWI (e.g., Barwood et al., 2006, 2007, 2013, 2014). Indeed, it has been shown that there are salient moderating influences on the extent of the CSR which are, at least in part, caused by high in contrast to low levels of anxiety (Glaser et al., 1959; Barwood et al., 2017). It has also been shown that components of the CSR can be influenced positively by psychological training thereby inducing an 80% improvement in maximal breath hold time on CWI (Barwood et al., 2006). Moreover, familiarity with the immersion scenario, thereby reducing the associated anxiety with immersion also has a beneficial effect. We showed that repeatedly experiencing the immersion sequence (i.e., repeated thermoneutral water immersion; 35°C) in the absence of a repeated cold-water stimulus leads to a small but significant reduction in respiratory tidal volume on subsequent CWI (Barwood et al., 2014). Accordingly, we concluded that repeated immersion in thermoneutral water induces a perceptual habituation of the threat posed by imminent immersion and this confers some benefit even when the water temperature is cold. Most recently we have shown that the concurrent experience of high levels of acute anxiety throughout a series of habituation immersions prevents or delays CSR habituation (Barwood et al., 2017). We suggested that the concomitant experience of anxiety disinhibits the transmission of thermal afferent information such that it magnifies the CSR response or prevents habituation; a mechanism first suggested by Glaser et al. (1959). Most importantly we suggested that the high levels of anxiety prevented or delayed the learned control of ventilation that we believe occurs during habituation since ventilation is under greater voluntary control than cardiac components (Barwood et al., 2017). Given that respiratory impairment is the primary threat to otherwise healthy individuals on CWI (Tipton, 2003), the consequences of impaired respiratory control caused by high anxiety levels in the emergency scenario may increase the risk of death by drowning.

Clearly the valence of the psychological experience prior to and during CWI is a potential driver of the physiological response that is seen; it is no longer appropriate to consider the CSR as solely a physiological phenomenon. The observations above question the reliability and potential practical value of inducing habituation to defend against the CSR in the emergency scenario. They may suggest that anxiety levels could underpin at least some of the variation in the CSR that is evident before and after habituation and suggest that the CSR should be considered as an integrative psychophysiological experience. If the extent of anxiety is an important mediator in the resultant CSR then the CSR should be altered by the experience of high(er) by contrast to low(er) levels of anxiety (Barwood et al., 2017).

Accordingly, from our database of recent immersions (*n* = 48), the present study examined the predictive relationship that prior ratings of acute anxiety have with components of the CSR on the first minute of immersion; the period widely accepted as critical in determining survival chances and thought to be under minimal voluntary influence (Tipton, 1989). Subsequently, we examined whether the anxiety we recorded during immersion correlated with components of the CSR before and after habituation of the CSR. Lastly, we examined whether these relationships changed when we experimentally induced increases in anxiety when contrasted to low(er) anxiety levels. We tested the experimental hypothesis that, if anxiety levels were an important mediator of the CSR, they would predict components of the CSR (H_1_). Secondly, we hypothesized that prior to a series of habituation immersions (i.e., on the first CWI) high levels of anxiety would predict the cardiac component of the CSR as learned control of ventilation was yet to occur (H_2_). Subsequently, we hypothesized that after repeated CWIs low(er) levels of anxiety would predict respiratory components of the CSR as low levels of anxiety would be permissive of voluntary respiratory control whereas high(er) levels of acute anxiety would predict cardiac components of the CSR which are under lesser voluntary control; thereby inferring the directional nature of the anxiety response.

## Materials and Methods

### Participants

The University of Portsmouth Science Faculty Research Ethics Committee provided ethical approval for the studies from which the data are drawn. The study was conducted in accordance with the Declaration of Helsinki and all participants provided written informed consent following a verbal and written briefing. The onward use of data, as in the case in the present analysis, was included in the consent procedure. The participants were non-smokers and were not cold water habituated, i.e., had not been exposed to cold water in the preceding year. They abstained from alcohol and caffeine consumption for 24 h before each test and from undertaking any exercise on the day of the test. The participants included in the present tests were pooled from our previous studies (Barwood et al., 2013, 2014, 2017) culminating in a maximum of 48 participants to choose from. To address our research questions we considered two cohorts; some of the participants were common between cohorts. Cohort 1 included forty-eight unhabituated participants (34 male, 14 female; mean[SD] age 20[2] years, height 1.75[0.1]m, mass 76.2[16.7]kg); these data were used to examine the relationships between the anxiety ratings and the CSR before habituation (i.e., during one of their first two immersions and when anxiety levels were not manipulated). Cohort 2 included 25 (16 male, 9 female; age 20[2] years, height 1.75[0.1]m, mass 77.9[17.2]kg) participants who completed further CWIs.

### Experimental Design

**FIGURE 1:**
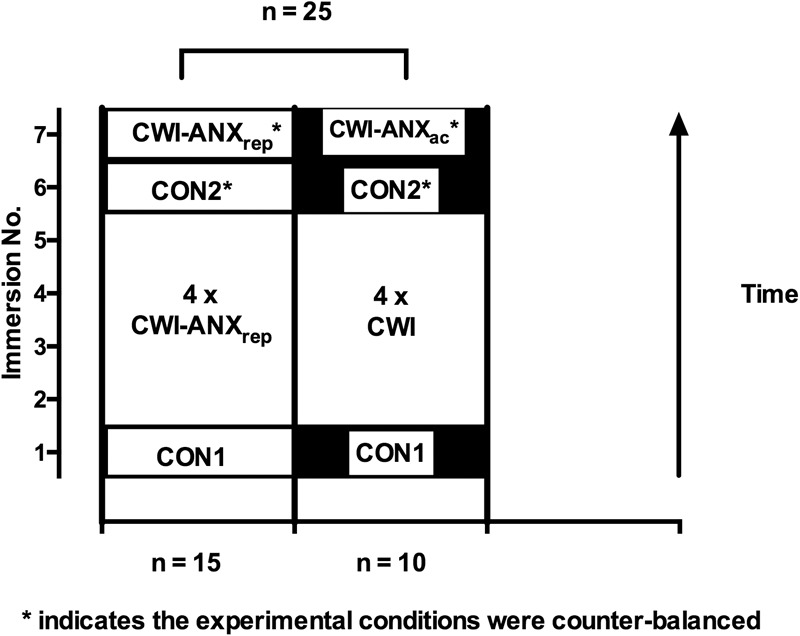
Order of experimental conditions and manipulations for participants in cohort 2; note: the participants were combined from two studies to make a total of 25 participants for analysis after the habituation immersions were complete.

Participants were recruited on the basis of undertaking a within participant, repeated measures, experimental design and acted as their own control; the experimental designs and manipulations in the respective studies are reported in detail elsewhere (Barwood et al., 2013, 2014, 2017). Briefly, all participants completed an initial CWI where anxiety levels were not manipulated but were expected to be naturally high due to novelty and unfamiliarity with CWI; forming cohort 1 (CON1; *n* = 48). In two of our four previous studies, participants underwent four further CWIs which would be sufficient to induce a habituation forming cohort 2 (see **Figure 1**; *n* = 25); albeit with different manipulations of anxiety levels during these immersions. Participants in cohort 2 completed two further counter-balanced CWIs during which time their anxiety levels were manipulated to be increased (CWI-ANX_ac_ or CWI-ANX_rep_ described as CWI-ANX hereafter) or were not manipulated (CON2). The order of CWIs and manipulations of anxiety therein are described in **Figure 1**. All immersions included in the present analysis were conducted at the same time of day within-participant.

### Immersion Protocol

Following arrival at the Extreme Environments Laboratory, each participant’s height (m) and mass (kg) was recorded using a stadiometer (Bodycare Stadiometer, Leicester, United Kingdom) and calibrated weighing scales (OHAUS digital weighing scales, Parsippany, NJ, United States). Each participant changed into their swimming costume. Males wore swimming trunks and females wore a swimsuit; the same swimming costume was worn by each participant on each occasion. Participants were then instrumented with a 3-lead ECG (HME Lifepulse, England) and entered an ambient temperature (T_a_) controlled laboratory. They sat on an immersion chair attached to an electrical winch (CPM, F1-8; 2-8; 5-4, Yale, Shropshire, United Kingdom) with a seat belt fastened around their waist to counteract buoyancy on immersion. The participant inserted a two-way mouthpiece (Harvard, United States) and attached a nose clip. The mouthpiece was connected to a spirometer (spirometric transducer module, KL Eng. Co, Northridge, CA, United States) by respiratory tubing in order to measure the respiratory responses to immersion. The participant was winched above the immersion tank to rest for 1-min. Thirty seconds into the 1-min rest period participants provided a rating of their state anxiety on a visual analog scale (Cella and Perry, 1986); they were familiarized with the scale in advance of the study. Toward the end of the 1-min period a 10-s verbal countdown preceded the participant being lowered at a reproducible rate (8 m min^-1^) until immersed to the clavicle in stirred water. Participants remained seated and with their limbs stationary during the immersion. After 1, 3, 5, and 7-min of immersion they again reported their anxiety rating, following which they were winched from the immersion tank. They then had a hot shower, re-dressed and left the laboratory. Each immersion was standardized immersing the participant to the same depth, at the same rate and in to stirred 15°C cold water.

### Anxiety Manipulations

In cohort 2 (i.e., see **Figure 1**), in either their penultimate or final immersion the participants were told by an independent researcher 2 min prior to immersion that the water temperature would be 5°C colder than their first immersion but in reality it was unchanged. This was achieved by slightly different means (i.e., perceived 1°C reductions across 4 immersions or one perceived 5°C reduction) to meet the experimental aims of the particular study (see Barwood et al., 2013, 2017); the cohorts are being grouped here to improve the statistical power of our observations.

### Measurements

Water temperature (T_w_) and T_a_ were measured and recorded using a calibrated thermistor [Grant Instruments (Cambridge) Ltd, Shepreth, United Kingdom) secured to the wall of the immersion tank and a Wet Bulb Globe Thermometer station respectively, both attached to a data logger [1000 series, Squirrel Data Logger, Grant Instruments (Cambridge) Ltd, Shepreth, United Kingdom]. Average T_w_ was closely matched within participant (±0.2°C) between CON1, and the sixth and seventh immersions (i.e., CON2, CWI-ANX). During the habituation immersions (i.e., immersions 2–5) the T_w_ was ± 0.5°C of 15°C.

### Cardiorespiratory Responses

The ECG and spirometer were interfaced with a digital data acquisition system (16SP PowerLab, Castle Hill, NSW, Australia) which captured data continuously throughout the rest and immersion periods. Chart analysis software (Chart version 6, AD Instruments Ltd, Oxford, United Kingdom) was used to automatically identify R-waves from the ECG and calculate cardiac frequency (*f*_c_); movement artifacts were visually identified and excluded from analysis. The spirometer was calibrated using a syringe of known volume (3 L syringe, Harvard Instruments, Harvard, United States). Respiratory frequency (*f*_R_) was recorded by Chart analysis software using auto-recognition of the peak after inspiration. The peak value after the onset of inspiration was recorded as tidal volume (*V_T_*) and multiplied by the calculated *f*_R_ to generate minute ventilation (*
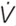
*_E_).

### Anxiety Perceptual Responses

**FIGURE 2:**
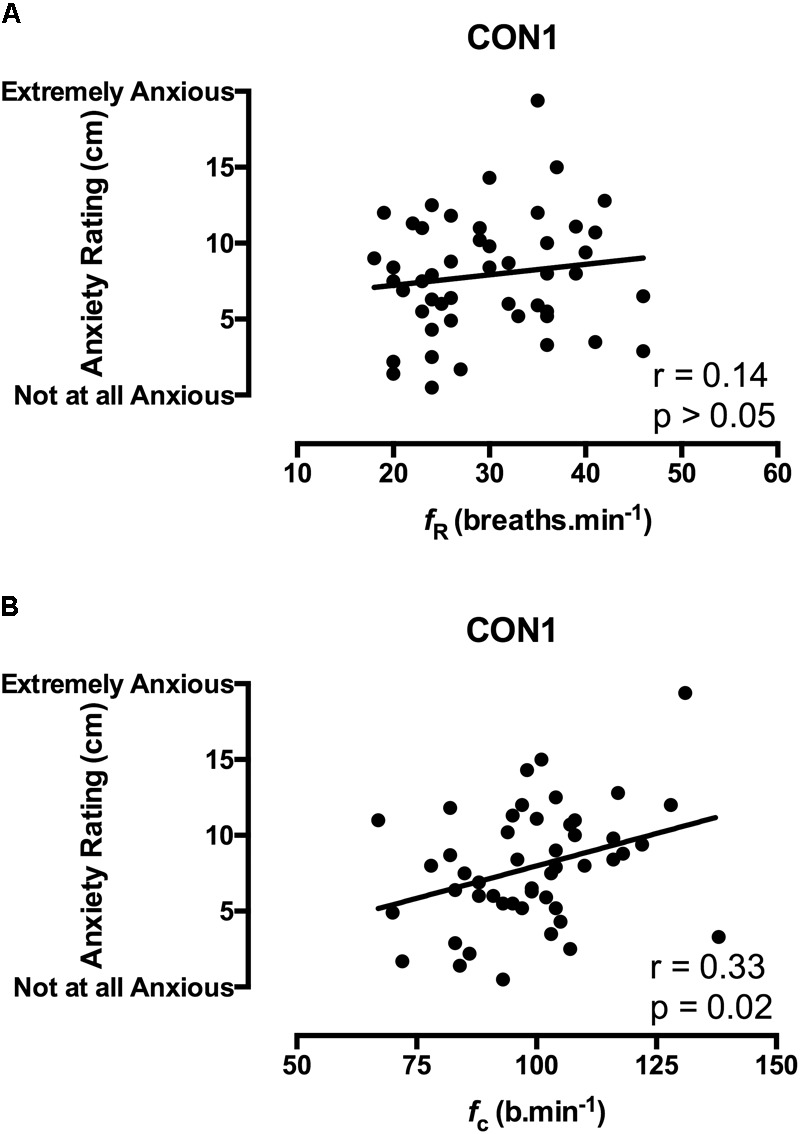
Correlation coefficients between acute anxiety ratings in the 1st minute of immersion and the *f*_R_ **(A)** and *f*_c_ **(B)** components of the CSR in unhabituated participants (*n* = 48).

**FIGURE 3:**
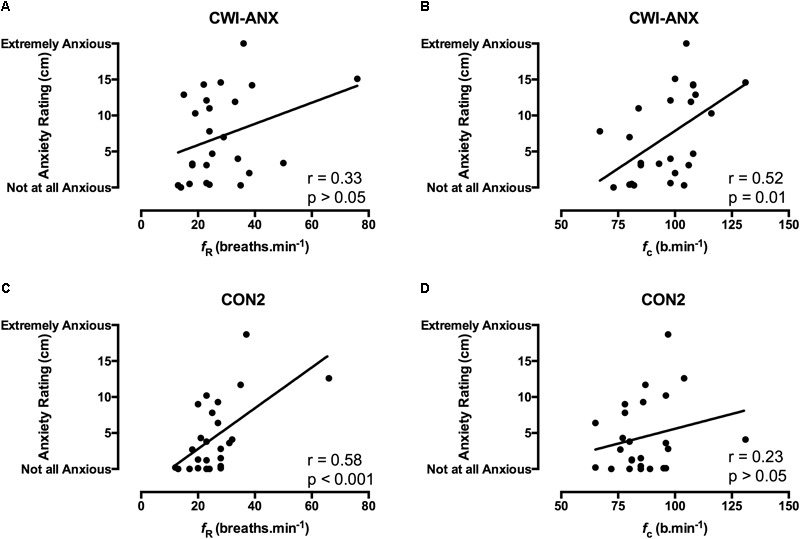
Correlation coefficients between acute anxiety ratings in the 1st minute of immersion and the *f*_R_ and *f*_c_ components of the CSR in habituated participants where anxiety levels were experimentally increased (CWI-ANX; **A,B**; *n* = 25) and anxiety levels were not manipulated (CON2; panels **C,D**; *n* = 25).

The state anxiety response to immersion was quantified using a 20 cm visual analogue scale (VAS) with descriptive phrases ranging from 0 cm (*not at all anxious)* to 20 cm (*extremely anxious*; Cella and Perry, 1986. Participants reported their anxiety by drawing a horizontal line on the vertical scale (see example in **Figures 2, 3**, y axes) that corresponded to their feeling of anxiety with the distance between points in centimeters providing a numerical value for the measure.

### Statistical Analysis

In order to examine the possible predictive relationship between *prior* anxiety levels on the resultant CSR *during* immersion, stepwise multiple regression analyses were undertaken between the anxiety rating recorded prior to immersion and the components of the CSR recorded in the first minute of immersion (i.e., the most life threatening period of acute immersion when the CSR peaks); comparisons were made to 1-min averages for *f*_c_, *f*_R_, *V_T_* and *
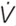
*_E_. This analysis was also undertaken for cohort 2 for immersions 6 and 7; i.e., the counter-balanced immersions after habituation when anxiety levels were manipulated to be high(er) or low(er).

In order to examine how the relationship between anxiety rating and components of the CSR developed throughout each immersion before and after completion of the habituation immersions, Pearson’s moment correlation coefficients were calculated across the 1st, 3rd, 5th, and 7th minutes of CON1, CON2 and CWI-ANX immersions. Analyses were undertaken using SPSS *v*22 and Prism 6.0 to an alpha level of 0.05.

## Results

### Multiple Regression Analysis

Anxiety rating prior to immersion was predictive (*p* < 0.05). The *f*_c_ component of the CSR was predicted in the unhabituated cohort (CON1; *r* = 0.536, *r*^2^= 0.190, *n* = 48). After habituation immersions the prior anxiety rating predicted different components of the CSR depending on whether anxiety rating was high, where *f*_c_ was again predicted in the CWI-ANX condition (*r* = 0.518, *r*^2^= 0.197, *n* = 25) or low where *f*_R_ was predicted in CON2 (*r* = 0.566, *r*^2^= 0.320, *n* = 25). Collectively, the anxiety rating prior to immersion predicted different components of the CSR before and after habituation immersions had taken place dependent upon whether anxiety ratings were high or low. In higher anxiety conditions (e.g., CWI-ANX and CON1 where in the latter case anxiety rating was high due to novelty), the anxiety rating predicted the *f*_c_ component of the CSR. When anxiety levels were low the anxiety rating predicted the *f*_R_ component of the CSR. The strength of the predictive relationship was far stronger when anxiety levels were low.

### Correlation Analysis

Correlation data support the directional relationships suggested by the regression model. In CON1 when anxiety levels were high the anxiety rating was correlated with *f*_c_ but not *f*_R_ in the 1^st^ minute of immersion (see **Figures 2A,B**). In the 3rd and 5th minute of immersion the anxiety rating was correlated with *f*_R_; see **Table 1**. An identical set of results was evident after habituation immersions (see **Figures 3A,B** for 1st minute data) when anxiety levels were manipulated to be higher (i.e., CWI-ANX) although this did not extend in to the 5th minute of immersion. Collectively the *r*-values indicated weak to moderate strength relationships (*r* = 0.29–0.52); see **Table 2**.

**Table 1:** Correlation between acute anxiety rating and components of the CSR in the 1st, 3rd, 5th, and 7th minutes of the first immersion (*n* = 48); ^∗^denotes significant correlation (*p* < 0.05).

		*CSR Component*			
Immersion period	Correlation result	*f*_R_ (breaths⋅min^-1^)	*f*_c_ (bt⋅min^-1^)	*V_T_* (L⋅min^-1^)	* 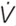 *_E_ (L⋅min^-1^)
1st min	*r*-value	0.14	0.33	-0.10	-0.02
	*p*-value	0.36	0.02*	0.52	0.89
3rd min	*r* value	0.30	0.24	0.09	0.28
	*p*-value	0.04*	0.10	0.53	0.06
5th min	*r*-value	0.29	0.18	0.03	0.21
	*p*-value	0.04*	0.21	0.84	0.16
7th min	*r*-value	0.10	0.26	0.25	0.32
	*p*-value	0.50	0.08	0.09	0.03*

**Table 2:** Correlation between acute anxiety rating and components of the CSR in the 1st, 3rd, 5th, and 7th minutes of the anxiety inducing immersions (i.e., immersion 6 or 7; CWI-ANX) after habituation immersions (*n* = 25); ^∗^denotes significant correlation (*p* < 0.05).

		*CSR component*			
Immersion period	Correlation result	*f*_R_ (breaths⋅min^-1^)	*f*_c_ (bt⋅min^-1^)	*V_T_* (L⋅min^-1^)	* 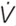 *_E_ (L⋅min^-1^)
1st min	*r*-value	0.33	0.52	-0.25	0.05
	*p*-value	0.11	0.01*	0.23	0.82
3rd min	*r*-value	0.40	0.28	-0.28	-0.01
	*p*-value	0.04*	0.18	0.18	0.97
5th min	*r*-value	0.32	0.25	-0.01	0.18
	*p*-value	0.11	0.22	0.97	0.40
7th min	*r*-value	0.33	0.11	-0.01	0.24
	*p*-value	0.11	0.61	0.96	0.25

*In contrast, in the 1st minute of the CON2 (*n* = 25) immersion acute anxiety levels were positively correlated with *f*_*R*_ but not *f*_*c*_ (see **Figures 3C,D**); a relationship also evident in the 5th minute of immersion. Anxiety ratings were correlated with the *f*_*c*_ component of the CSR in the 3rd minute of immersion; see **Table 3**. Collectively the *r*-values more consistently indicated moderate strength relationships (*r* = 0.40–0.58).*

## Discussion

We hypothesized that if anxiety levels were an important mediator of the CSR they would predict components of the CSR prior to immersion; we support our hypothesis (H_1_). Our data support the idea that high levels of anxiety, which occurred instinctively before an initial immersion (i.e., CON1) and were manipulated to be increased on one occasion after habituation immersions (i.e., CWI-ANX), would be predictive of the cardiac component of the CSR. By contrast, when anxiety levels were allowed to fall after habituation immersions, the low(er) levels of anxiety were predictive of the respiratory frequency component of the CSR; this finding infers a mediating role for anxiety level. Given that first minute of immersion is the most life-threatening as it is when the CSR peaks (Tipton, 1989), it seems prudent that interventions to aid those at risk of CWI should additionally aim to reduce anxiety prior to and on immersion.

We also examined the relationships that were evident between components of the CSR and acute anxiety ratings *during* immersions. The results in the first minute of immersion support the predictive relationship evident with our regression model with high levels of anxiety associated with priming the cardiac component of the CSR and low(er) levels associated with driving the respiratory frequency component of the response. The clarity of this relationship is not sustained in to the 3rd, 5th, and 7th minutes of immersion with the resultant effect of high anxiety levels not necessarily exclusively associated with one or other component of the CSR thereafter (see **Tables 1, 2**). However, it is clear that the significant relationships seen with high(er) levels of anxiety (i.e., CON1 and CWI-ANX) by contrast to low(er) levels (i.e., CON2) temporally oppose one another (view **Tables 1–3**). Moreover, when anxiety levels were not manipulated to be increased after habituation immersions (i.e., CON2), the anxiety rating is less variable, more consistently and strongly associated with the respiratory frequency component (i.e., it approached a significant relationship in the 3rd minute of immersion and was significant in the 1st and 5th). Although correlation does not confer causation, the observation from our regression model provides some quantifiable evidence of this effect with approximately 20% of the variance in *f*_c_ explained when anxiety levels are high whereas 32% of the variance is explained by *f*_R_ when anxiety levels are allowed to fall. Clearly, some of the evident variation in CSR seen in previous studies must be accounted for by differing levels of anxiety about impending immersion and it is also clear that the predictability of the CSR is improved when anxiety levels are lowered (see **Figures 2, 3**); a further reason to target lowering anxiety levels by way of preparatory training for those at risk. It is also possible that this benefit would extend to improving breath-hold time on immersion which may be a requirement in some situations (e.g., ditched helicopter egress).

**Table 3:** Correlation between acute anxiety rating and components of the CSR in the 1st, 3rd, 5th, and 7th minutes of the control immersion (i.e., immersion 6 or 7; CON2) after habituation immersions (n = 25); ^∗^denotes significant correlation (*p* < 0.05).

		*CSR Component*			
Immersion period	Correlation result	*f*_R_ (breaths⋅min^-1^)	*f*_c_ (bt⋅min^-1^)	*V_T_* (L⋅min^-1^)	* 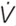 *_E_ (L⋅min^-1^)
1st min	*r*-value	0.58	0.23	-0.09	0.45
	*p*-value	0.00*	0.28	0.68	0.02
3rd min	*r*-value	0.36	0.40	0.06	0.14
	*p*-value	0.08	0.05*	0.78	0.51
5th min	*r*-value	0.49	0.37	0.16	0.49
	*p*-value	0.01*	0.07	0.44	0.01
7th min	*r*-value	0.37	0.21	-0.09	0.11
	*p*-value	0.07	0.32	0.67	0.61

The protective benefit that habituation would provide in the real life scenario is now questionable given that: we have shown previously that habituation is prevented/delayed when anxiety levels are not concurrently reduced during repeated immersions (Barwood et al., 2017); habituation is partially reversed (cardiac component only) when subsequent high levels of anxiety are experienced (Barwood et al., 2013); conversely and as shown in the present study, low levels of anxiety which are less plausible in the emergency scenario are permissive of respiratory control. Consequently, we contend that the specificity of the habituation stimulus (also see Leblanc and Potvin, 1966) in reflecting the real-world scenario plays an important role in the response that is evoked. Therefore, our habituation techniques (e.g., survival training) in preparing at risk individuals should be as reflective of the true stimulus as is possible. The resultant effects of anxiety on different components of the CSR are also important given that the primary risk on accidental CWI is caused by a loss of respiratory control and the associated increased risk of aspirating water to the lungs (Tipton, 1989). It has been speculated that this mechanism accounts for a significantly larger proportion of sudden immersion deaths with the remainder (∼10%) accounted for by a sudden cardiac event (Tipton, 2003). Hence a significantly increased heart rate would be of a lesser concern than a raised respiratory rate on immersion in an otherwise healthy individual. Our previous finding in unhabituated participants that high levels of anxiety augment both the ventilatory and cardiac components of the CSR probably applies to the majority of those who are accidentally immersed (Barwood et al., 2013). Collectively, this suggests that anxiety level should be added to our future measurements of the CSR and an integrated psychophysiological model is required to consider the multifactorial CSR drivers. Accordingly we offer some novel insights on potential neuroanatomical, perceptual and attentional components that may be responsible for our observations.

### Neuroanatomy of the Stress Response

Concurrent to an activated thermal neural network, we suggest that emotion (e.g., panic and anxiety), attentional processing and behavior could all influence respiratory motor output as has been observed in studies examining respiratory responses to panic and fear (Masaoka and Homma, 2001). Areas of the hypothalamus, forebrain, limbic and cortical structures have been implicated in the biological systems that process information from the external environment resulting in stimulation of spinal respiratory motor neurones thereby increasing respiratory rate (*f*_R_) but not ventilatory depth (*V_T_*; Masaoka and Homma, 2001, 2004); we also saw no change in *V_T_*. These anatomy also share a common anatomical connection with the spinal lamina I neurons which convey the thermoafferent volley triggered by sudden skin cooling (see Craig, 2002) which also evoke the CSR (for reviews see Tipton, 1989; Datta and Tipton, 2006; Tipton et al., 2017). Small afferent Lamina I neurons are part of the Lamina I spinothalamocortical pathway that relay afferent information to the main homeostatic integration sites in the brainstem (Todd et al., 2005). The brainstem projects to the insula cortex, which also receives afferent input from the somatosensory cortex (Craig, 2002) both of which we suggest are important in producing the sensory experiences prior to and on immersion. When immersion is planned as in the present studies we suggest that, based on the ideas of Craig et al. (2000) model of interoception, the dorsolateral (DL)PFC provides the insula with corollary discharge which predicts the expected sensory consequences of the immersion. On receiving the afferent feedback the insula compares the predications with the actual afferent information in order to generate a current awareness state (Craig, 2002;Gu et al., 2013). We speculate that discrepancies between the actual and expected afferent signals, magnified by our high compared to low anxiety conditions, may produce an altered physiological response and differential activation of *f*_c_ in the high anxiety condition and *f*_R_ in the low anxiety condition. Craig (2002) and others (Medford et al., 2010) suggest that other brain areas including the anterior cingulate cortex (ACC), medial (m)PFC and DLPFC are important in generating this awareness state with the insula cortex and ACC also including neural connections to the amygdala (Shi and Cassell, 1998) which may account in part for the emotional effects found in our previous research (Barwood et al., 2013, 2017).

### Thermal and Stress Habituation

In understanding the likely interaction between the repeated thermal and perceptual stress induced by our experiments it is important to consider the central site and mechanism by which thermal stimulation may be consolidated and habituation mediated. Historically, studies of CWI habituation have linked the frontal cortex to habituation of the CSR (Glaser and Griffin, 1962; Griffin, 1963). More recently, the sub-division of the cerebral cortex, the prefrontal cortex (PFC) has been linked with behavioral and cognitive flexibility (i.e., the process by which environmental feedback is used to modify behavior) the normal function of which is absent in stress related disorders (Campeau et al., 2011). Animal studies involving cold stress have been shown to impact the orbitofrontal subregion of the PFC whereas the prelimbic and infralimbic cortices are more specifically linked to responses to chronic psychological stressors (Campeau et al., 2011). In line with the mechanism outlined above, the spinal Lamina I pathway includes projections to the orbitofrontal sub-region of the mPFC, which consists of Brodmann’s areas 10, 11, and 47 and is thought to play a major role in cold stress interpretation and the associated hedonic tone (i.e., pleasant or unpleasant nature) of a given event (Kringelbach, 2005). This is consistent with the idea that the orbitofrontal cortex encodes the outcome expectations of a given situation (Schoenbaum et al., 2009) which were manipulated in the present study in the high compared to low anxiety conditions. This region has also been found to play a major role in risk avoidance (Brown and Braver, 2007). The mPFC, in turn, sends ascending projections to the DLPFC which, following habituation, is responsible for re-configuration of predictions of sensory effects that it will pass to the insula cortex in the form of corollary discharge (Kringelbach, 2005) during future immersion situations.

### Perceptual and Attentional Demands of CWI

We have previously hypothesized that a model of stress and coping may prevail in the emergency scenario that may ultimately increase or decrease the perceptual component and resultant anxiety response depending upon the primary and secondary appraisal of the stimulus (Barwood et al., 2013, 2014, 2017) thereby stimulating or mitigating the activity of the multiple neural networks involved on CWI. Accordingly, anxiety level may be increased if a victim is confronted with a highly novel and important stimulus (primary appraisal) which may be compounded by the perception that coping resources are limited or absent to deal with this situation (i.e., secondary appraisal) thereby resulting in a perceived threat (Lazarus and Folkman, 1984). Anxiety levels may be reduced if the stimulus were appraised as familiar, consequently less important and when accompanied by a perceived high level of coping resource, as might be the case in those who receive survival training or basic survival skills for the immersion scenario. Models of attentional processing are required to explain the real time attentional demands of accidental immersion given its critical nature. One proposition is that, when under duress, the brain has a finite processing capability that is narrowed by increased arousal levels as might be expected in the emergency scenario (Rejeski and Ribsl, 1980). Evidently, filtering multiple relevant environmental and behavioral cues (i.e., ‘floating first,’ waiting for CSR to subside, keeping the airway above the water; Barwood et al., 2011, 2016) whilst ignoring the irrelevant cues may not be feasible when attentional capacity is limited. Similarly, a processing efficiency theory would contend that, under stress, working memory is taken up with worry, anxiety and intrusive thoughts that consume limited working memory capacity and deny ressources for processing important task-relevant information (Eysenck and Calvo, 1992). Both theories suggest that processing capacity would be limited in the acute, accidental immersion scenario whereby survival training or basic survival skills could be used to guide behavior at a time when attentional demand is high and the resultant decisions are critical. The concurrent decline in cerebral blood flow on immersion (Mantoni et al., 2007; Button et al., 2015) may compound any decrement to cognitive performance and increase the risk of drowning. One such possibility to improve any cognitive decrement on accidental immersion is to integrate a form of psychological skills training (PST) technique into survival training techniques or safety behavior messages. Such techniques have been shown to extend maximal breath-hold duration by up 80% on CWI in unhabituated participants (Barwood et al., 2006) and 120% (of that seen in air) in habituated participants (Barwood et al., 2007). Safety statements such as ‘float first’ include important procedural and environmental behavioral cues, similar to the embedded cognitive principles of PST, that convey important information in a succinct manner.

### Limitations

Clearly it is not ideal that we are considering the resultant effects of high(er) compared to low(er) acute anxiety after habituation immersions which included different experimental manipulations. Observations from animal studies (i.e., Kelly et al., 2011) may suggest that participants experiencing CWI concurrent to psychological stress (i.e., CWI-ANX_rep_ see **Figure 1**) would experience independent, and potentially additive, serotonergic drivers of the stress response resulting in different or absent habituation (see Barwood et al., 2017). Yet, irrespective of whether anxiety is manipulated or not during habituation immersions, it is experimentally very difficult to entirely remove the anxiety associated with impending and ensuing immersion. The tentative findings we show here with our combined analysis require subsequent verification; as is the case with all novel experimental findings. Yet we acknowledge that the neural mechanisms we outline above may have been stimulated differently in some of our participants *during* the habituation immersions despite experiencing an identical thermal stimulus. However, most importantly, the high vs. low level anxiety conditions we feature in the present study clearly showed a separation in the predictive and related components of the CSR despite the manipulations in the habituation immersions; hence we have been able to interrogate our hypotheses.

The water temperature used in the present experiments and our indices of the CSR may allow a mediating role for acute anxiety when none would be evident. In the case of our CSR indices, it is possible that a better index of respiratory drive than the one used here (e.g., mouth occlusion pressure at 100 ms of inspiration; P0.1) would share a different relationship with anxiety level than respiratory frequency does. P0.1 has been shown to more closely track the thermally induced neural drive to breathe during immersion whilst ventilation was shown to plateau (Mekjavic et al., 1987; Burke and Mekjavic, 1991). Hence the contribution of anxiety may not be truly reflected in our chosen index of respiratory drive after ventilation has plateaued. Given that the most critical period of the CSR is the first 60–90 s during which ventilation has yet to plateau, the issue of misrepresentation of respiratory drive by respiratory frequency is more likely to be a factor later in the 7-min immersion period we have studied here. In the case of water temperature, the CSR is suggested to be maximally evoked at water temperatures between 5°C and 15°C (Goode et al., 1975; Tipton et al., 1991) although nocioceptors may be activated at temperatures below 10°C. Indeed, studies that examine the cardiorespiratory response to pain in anesthetized patients (Eger et al., 1972; Borgbjerg et al., 1995) and decerebrate cats (Waldrop et al., 1984) suggest that neural pain networks transmit nocioceptive information to the bulbar respiratory nuclei without involving higher cortical centers. Hence, nocioceptors and thermoreceptors share a relatively direct and uncomplicated neural pathway to the respiratory centers the former of which may have an additive effect on the CSR that is seen. However, it must be noted that the *anticipation* of pain does stimulate higher cortical centers and result in an increase in respiratory frequency (Willer, 1975). In the present study, a water temperature of 15°C reduces the possibility that pain networks are also driving the response. A theoretical maximum CSR must exist for each immersed individual and we speculate this lies at the saturation point of the sympathetic branch of the autonomic nervous system with thermal, pain and perceptual stimuli.

Lastly, the gender imbalance in favor of males may also contribute to our findings and studies sub-divided by gender may be worthy future line of enquiry. Similarly, the rate of immersion in the present study was carefully controlled but does not reflect the rapid rate that would be evident on accidental immersion. It is known that cutaneous thermoreceptors respond to the rate and summation of a thermal stimulation culminating in the centrally integrated thermoafferent signal (Hensel and Schafer, 1984). Clearly both the rate and the summation of thermoreceptor stimulation would be higher on falling in to water as opposed to staged immersion. Other researchers have achieved more ecologically valid means of water entry whilst studying the CSR in the laboratory (Croft et al., 2013) although our methods do demonstrate a consistently administered and controlled thermal stimulus.

## Conclusion

Acute anxiety prior to immersion predicts different components of the CSR on immersion before and after habituation. This suggests that safety training and behavioral advice to survive accidental CWI should consider interventions that can also reduce acute anxiety about impending immersion particularly in at risk individuals. The CSR should be considered as an integrative psychophysiological response which may include the activation of multiple neural networks. We offer a new integrated model of the CSR and CSR habituation to explain our observations. Further studies are required to test this model.

## Author Contributions

All authors listed have made a substantial, direct and intellectual contribution to the work, and approved it for publication.

## Conflict of Interest Statement

The authors declare that the research was conducted in the absence of any commercial or financial relationships that could be construed as a potential conflict of interest.
